# The availability of snack food displays that may trigger impulse purchases in Melbourne supermarkets

**DOI:** 10.1186/1471-2458-12-194

**Published:** 2012-03-15

**Authors:** Lukar E Thornton, Adrian J Cameron, Sarah A McNaughton, Anthony Worsley, David A Crawford

**Affiliations:** 1 Centre for Physical Activity and Nutrition (C-PAN) Research, School of Exercise and Nutrition Sciences, Deakin University, Melbourne Burwood Campus, 221 Burwood Highway, Burwood, Vic 3125, Australia

**Keywords:** Food environment, Snack food, Supermarkets, Area-level disadvantage

## Abstract

**Background:**

Supermarkets play a major role in influencing the food purchasing behaviours of most households. Snack food exposures within these stores may contribute to higher levels of consumption and ultimately to increasing levels of obesity, particularly within socioeconomically disadvantaged neighbourhoods. We aimed to examine the availability of snack food displays at checkouts, end-of-aisle displays and island displays in major supermarket chains in the least and most socioeconomically disadvantaged neighbourhoods of Melbourne.

**Methods:**

Within-store audits of 35 Melbourne supermarkets. Supermarkets were sampled from the least and most socioeconomically disadvantaged suburbs within 30 km of the Melbourne CBD. We measured the availability of crisps, chocolate, confectionery, and soft drinks (diet and regular) at the checkouts, in end-of-aisle displays, and in island bin displays.

**Results:**

Snack food displays were most prominent at checkouts with only five stores not having snack foods at 100% of their checkouts. Snack foods were also present at a number of end-of-aisle displays (at both the front (median 38%) and back (median 33%) of store), and in island bin displays (median number of island displays: 7; median total circumference of island displays: 19.4 metres). Chocolate items were the most common snack food item on display. There was no difference in the availability of these snack food displays by neighbourhood disadvantage.

**Conclusions:**

As a result of the high availability of snack food displays, exposure to snack foods is almost unavoidable in Melbourne supermarkets, regardless of levels of neighbourhood socioeconomic disadvantage. Results of this study could promote awareness of the prominence of unhealthy food items in chain-brand supermarkets outlets.

## Background

Consumption of unhealthy (energy-dense, nutrient poor) snack foods has become common-place in recent decades [1-3]. These consumption patterns are likely to be influenced by increased opportunities to purchase snack foods [4]. For instance, snack food can now be purchased in food stores [5,6], non-food stores [7] (e.g. pharmacies, gas stations) and at other common amenities (e.g. cinemas, transport termini).

Although the supermarket represents only one food shopping location, it is present in most urban geographic areas in Australia and is visited frequently by most of the population [8-10]. Therefore, the within-store supermarket environment is an important focal point for public health nutrition research. A recent audit of a large Melbourne supermarket found 1070 snack food items and 863 different beverages available, over 70% of which were considered inconsistent with a healthy diet [11]. Within supermarkets, snack foods are often displayed near the entrance and at checkouts [12-14] and promotions associated with such products are likely to be common [15]. The placement of snack foods at checkouts in 24 Melbourne supermarkets has been reported previously [16]. That study audited 257 checkouts with 87% and 80% displaying chocolate and other sweets, respectively. While prior studies have investigated snack food displays at checkouts, a key in-store location where retailers attempt to sell items likely to be purchased on impulse, these only represent a single point of exposure within supermarkets. Other displays such as those at the ends of aisles and in island bins may also trigger an impulsive choice prior to reaching the checkout.

Some research has suggested that supermarket stocking practices and store displays may vary according to the socioeconomic characteristics of the local area in which a store is located. For example, studies from the US have found fewer healthy choices available within-stores in more deprived neighbourhoods [17,18]. Our own recent analysis of shelf-space dedicated to snack food items and soft drinks showed that this did vary between supermarkets from the least and most disadvantaged neighbourhoods of Melbourne [34]. Other studies that have not demonstrated area-level socioeconomic differences in snack food exposure in supermarkets have been hampered by limited sample sizes or by crude in-store measurements [5,19]. Where observed, greater exposure to energy-dense snack foods in disadvantaged neighbourhoods could potentially promote socioeconomic variations in snack food purchasing.

Impulse food purchases (unplanned) have been shown to often be both unhealthy and heavily influenced by the presence of within-store displays and promotions [20,21]. Studies from Scotland [12] and Canada [13] highlight the effect that supermarket display strategies have on promoting sales of snack foods. An improved understanding of energy-dense snack foods in supermarkets may be used to lobby for a more health-promoting food shopping environment. We report here the findings of an investigation into the availability of crisps (potato chips), chocolate, confectionery and soft drinks (both diet (low energy) and regular) at checkouts, end-of-aisle displays and island bin displays within supermarkets in Melbourne, Australia, and whether these differed according to area-level socioeconomic disadvantage.

## Methods

### Sampling strategy

Supermarkets for this study were sampled from urban neighbourhoods (defined by suburb boundaries (mean population 9,280; mean area 7.8 km^2^)) within approximately 30 kilometres of the city centre of Melbourne, Australia. Each neighbourhood within this radius was ranked according to the Socio-economic Index for Areas (SEIFA) produced by the Australian Bureau of Statistics. We used the SEIFA Index of Relative Social Deprivation (IRSD) which takes into account factors from a range socioeconomic measures including income and education [22]. We extracted all suburbs from the highest and lowest quintiles of the IRSD and compiled a list of Coles and Woolworths supermarkets within the sampled suburbs. These supermarket chains account for approximately 80% of the market share in Australia [8]. The locations of all supermarket outlets were identified through company websites and other online directories (e.g. White Pages). After stratifying this list by level of suburb disadvantage and supermarket chain, forty-two supermarkets were randomly selected to survey. Our sample represents 35% and 50% of all Coles supermarkets in the most and least disadvantaged neighbourhoods, respectively, and 42% and 82% of all Woolworths supermarkets that are in the most and least disadvantaged neighbourhoods, respectively.

### Auditing process

Consent from store managers was obtained before taking any measurements within a store. The project proposal was assessed by a Human Research Ethics Advisor from the Office of Research Integrity at Deakin University who advised that ethics committee approval for the study was unnecessary because data collection did not involve personal disclosure. Consent to audit stores was received at 35/42 supermarkets (83.3%). Of the seven stores where consent was not gained, four (57.1%) were from the most disadvantaged suburbs.

The methods to measure snack foods at the checkouts, end-of-aisle displays and island displays were developed and pilot-tested in several supermarkets and two fieldwork staff were provided with written instructions and trained in supermarkets in the use of the audit tool. The audits were conducted in two time periods between September 2010 and November 2010 and between January 2011 and February 2011. This largely avoided the peak Christmas period where it was expected snack food displays may have been greater.

#### Checkouts and end-of-aisle displays

Auditors assessed whether snack foods were available at each of the store's checkouts and end-of-aisle displays at both the front (nearest to checkouts) and the back of the store. Using a checklist, the presence of each of the following items was recorded: 1) soft drink - regular; 2) soft drink -diet; 3) crisps (potato chips); 4) chocolate (either as chocolate bars, blocks, boxes or bags); 5) confectionery/lollies (excluding chewing gum). Multiple item types could be recorded for each checkout or end-of-aisle display. Results are reported as a percentage of the total number of checkouts or end-of-aisle displays (front and back reported separately) that displayed any snack food item and again for each item separately.

#### Island bin displays

Island bin displays are temporary displays within the store that often change and are used to display products that are on sale or as part of a promotion. Auditors recorded the number of non-fixed island displays that contained the five items listed above and the circumference of each island. The length of each side was recorded by either a measuring wheel or (for smaller island displays) by a hand-held tape measure. For island bins where more than one product type was included, the circumference for each product type was calculated as the circumference of the island divided by the number of product types present. The total circumference of the island displays were tallied for each item in each store.

#### Store size

The length (in metres) of each aisle within the supermarket was measured using a measuring wheel. Total store size was quantified as the sum of aisle length.

### Statistical analysis

Distribution graphs were plotted (using the user-written (N.J. Cox) stripplot command in Stata) that displays the percentage of each snack food item at supermarket checkouts and end-of-aisle displays (front and back) and the circumference (metres) of island bin displays. The median and interquartile ranges (IQR) for each snack food item were included on all figures. Pairplot graphs (created using the user-written (N.J. Cox) pairplot command in Stata) were used to show the difference in the percentages of front-of-aisle displays and back-of-aisle displays for each item in each store. Independent sample t-tests were used to compare differences in percentages of checkouts and end-of-aisle displays with snack food items between stores in neighbourhoods from the top and bottom quintiles of relative socioeconomic disadvantage. For island bin displays, the estimated marginal mean circumference for stores from the top and bottom quintiles of socioeconomic disadvantage was calculated using models that also included a term to adjust for total store size.

## Results

### Checkouts

**Figure 1:**
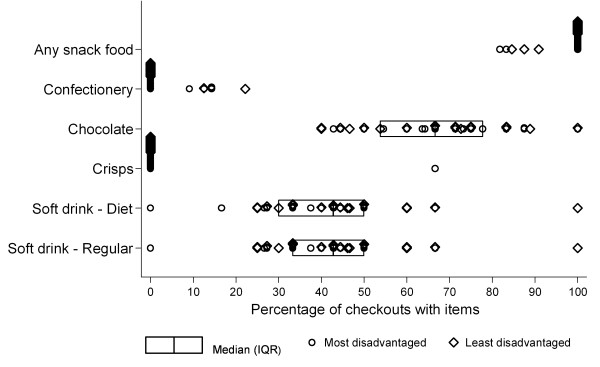
**Percentage of checkouts displaying snack foods within Melbourne supermarkets**.

Across the 35 surveyed supermarkets the number of checkouts in the stores varied between 4 and 20 (median 8, IQR 6-11). In all but five stores, at least one of the snack foods was displayed at every checkout. Of the remaining five stores, the lowest percentage of checkouts displaying any of the snack foods was 82%. The median percentage of checkouts that displayed soft drinks was just over 40%, with similar percentages observed for regular (median 43%, IQR 33%-50%) and diet soft drinks (median 43%, IQR 30%-50%) (Figure 1). Chocolate was the most common item of the snack foods assessed to be observed at the checkouts (median 66%, IQR 54%-78%) whilst very few checkouts had crisps or confectionery items available. The percentage of checkout displays containing each of soft drinks, crisps, chocolate, and confectionery were similar in stores from the least and most disadvantaged neighbourhoods (all p > 0.05).

### End-of-aisle displays

**Figure 2:**
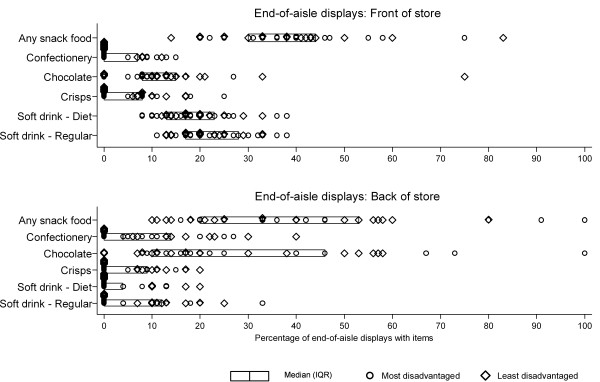
**Percentage of front-of-aisle and back-of-aisle displays with snack food present in Melbourne supermarkets**.

The median percentage of end-of-aisle displays containing snack food within a store was 38% (IQR 30% - 44%) for displays at the front of the store (adjacent to the checkouts) and 33% (IQR 20% - 53%) for displays at the back of the store. The median percentage of front-of-aisle displays with soft drinks was 22% for regular varieties (IQR 17% - 30%) and 17% for diet varieties (IQR 13% - 23%) (Figure 2). Few front-of-aisle displays included crisps (median 6%, IQR 0%-8%) or chocolate (median 10%, IQR 8%-15%) whilst confectionery was almost never displayed at the front-of-aisles (median 0%, IQR 0% - 5%). At least half of the stores did not display regular soft drink (median 0%, IQR 0% - 13%), diet soft drinks (median 0%, IQR 0% - 4%) or crisps (median 0%, IQR 0% - 9%) at any of their back-of-aisle displays. The median percentage of back-of-aisle displays with chocolate available was 18% (IQR 11% - 46%) and 4% for confectionery (IQR 0% - 14%).

**Figure 3:**
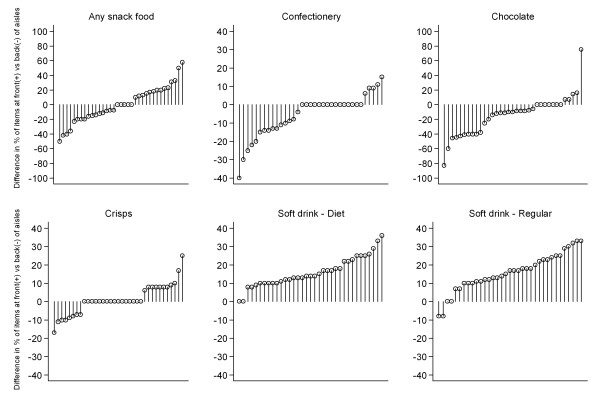
**Difference in the percentage of snack foods displayed in front compared with back of aisle displays in Melbourne supermarkets**.

Figure 3 displays the percentage of snack food items at the front-of-aisle displays and back-of-aisle displays within stores. For all snack food items combined, almost equal numbers of stores had the majority of snack food in front-of-aisle or back-of-aisle displays. Front-of-aisle displays more often contained soft drink in comparison with back-of-aisle displays, however the reverse was true for chocolate and confectionery (Figure 3). No significant variation in the percentage of end-of-aisle displays containing soft drinks, crisps, chocolate or confectionery was observed according to level of neighbourhood disadvantage (all p > 0.05).

### Island bin displays

**Figure 4:**
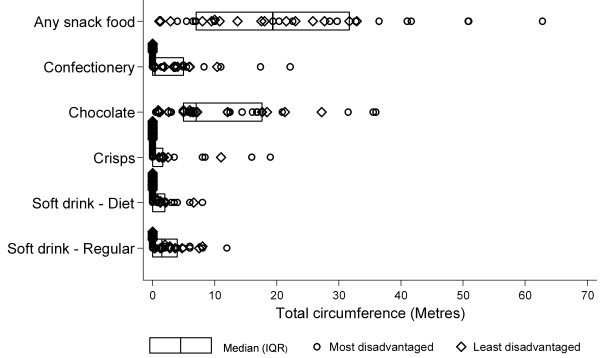
**Circumference (metres) of island bin displays containing snack foods in Melbourne supermarkets**.

The sampled supermarkets contained a median of 7 (IQR 3-13) island bin displays with these more often containing chocolate (median 4, IQR 2 - 7) (Table 1). These island displays equated to a median of 19.4 metres (IQR 7.0 m - 31.7 m) of snack food displays in addition to that already present in fixed displays in the supermarkets shelves (Figure 4). Five of the stores audited had an additional 40 metres or more of snack food displayed in island bins which is roughly equivalent to an additional 1.7 extra supermarket aisles (mean aisle length 23.0 m in audited stores) dedicated to snack food. Chocolate was the most commonly represented item of the snack foods assessed in island bin displays (median circumference 7.0 m, IQR 5.0 m - 17.6 m). Total circumference of island bin displays containing snack food was larger in stores in the most disadvantaged areas (p = 0.030). This circumference remained larger in stores in the most disadvantaged areas after adjustment for total stores size but was no longer statistically significant (p = 0.161) (results not shown).

**Table 1:** Median number of island bin displays containing snack foods within Melbourne supermarkets

Item	Number of island bins item appears in Median (IQR)
Any snack food	7 (3, 13)
Confectionery	1 (0, 3)
Chocolate	4 (2, 7)
Crisps	0 (0, 1)
Soft drink - Diet	0 (0, 1)
Soft drink - Regular	1 (0, 2)

## Discussion

The results from this investigation demonstrate the ubiquitous exposure to displays of chocolate, crisps, confectionery and soft drinks within Melbourne supermarkets. Such displays increase the frequency in a shopping trip that supermarket customers are exposed to snacks foods within Melbourne supermarkets and are designed to increase impulse-driven purchases [12-14]. Within the studied supermarkets, we found chocolate was the most prominent snack food item on display, appearing at the majority of checkouts and most frequently in island bin displays. Interestingly, while chocolate was the most common snack food item at the back-of-aisle displays, soft drinks (both diet and regular) were more common at the front-of-aisle displays. It is likely that this is because chocolate is already offered at the checkouts located opposite to the front-of-aisle displays. Each of the displays investigated here are considered dynamic in nature and are essentially independent of static aisle shelf displays.

Within supermarkets, consumers make a number of unplanned purchasing decisions, some of which are impulsive [10,14]. Individuals who make unplanned and impulsive food choice are more likely to consume lower amounts of healthy foods such as fruits and vegetables [20] while those with an impulsive personality trait are more likely to overeat and be overweight [23]. Food retailers themselves recognise that snack foods such as confectionery are frequently bought impulsively [12]. Where hunger interacts with impulsiveness [21] or in-store marketing/promotions exist [24], the purchase of snack foods is likely to be higher. Consequently, the display of snack food items in supermarkets is not random and relies upon a profit driven approach [12,13]. By providing snack food at the checkouts and broadening the range of snack food displays in different parts of the stores, retailers maximise the opportunity to sell snack food items on impulse [12,13].

While previous research has demonstrated similarly high numbers of snack foods at supermarket checkouts [13,16], we have also reported other potential incidental exposures within the store. This is an important distinction as our results demonstrate that at least in those supermarkets examined, the combination of snack food available in end-of-aisle, checkout, and island bin displays, in addition to the regular position in the aisle shelves, means exposure to snack-food displays within supermarkets is almost unavoidable. What remains to be ascertained is how these exposures are likely to impact on food purchasing decisions and health. Shelf space dedicated to snack foods was reported to be unrelated to socioeconomic differences in snack food purchasing in Australia (although this study may have been underpowered to detect differences) [19] while another US study reported small positive correlations between shelf space with BMI [25]. However, in another study, a greater variety of snack food items in supermarkets was unrelated to snack food consumption [26], highlighting a need for further research.

The presence of supermarkets in a neighbourhood has been linked to healthier eating and a lower weight status [27,28] and they are also likely to provide local employment opportunities. Despite these potential community-level benefits, our findings raise important questions about the role of product availability and placement within supermarkets in promoting healthy eating behaviours. The level of supermarket exposure to energy-dense, nutrient-poor snack foods is at odds with what is required to prevent further escalation (or even reversal) of current high obesity rates. 'Parents Jury' campaigns in Australia [29] and the UK [30] have called for the removal of confectionery items from checkouts within supermarkets. In response, food retailers in the UK took one of three approaches. Some were proactive in removing confectionery from all checkouts; some offered specific confectionery-free checkouts; and some resisted all calls to remove confectionery from their checkouts [12]. Although the removal of confectionery from checkouts gives the impression of a win for public health advocates, the reality is that retailers often use other prime locations for snack food displays (including the end-of-aisles) and since the removal of snack foods from checkouts, some evidence suggests that sales in the form of multipacks actually increased [12].

By including supermarkets from least and most socioeconomically disadvantaged neighbourhoods, we were able to account for whether the displays were socioeconomically patterned however we found no evidence of this. One plausible explanation is that the nature of the displays presented in this study are more likely to be dictated by other market forces such as promotions by snack food companies and that any variation by area-level disadvantage is more likely to be observed in the regular aisle displays [34]. Further, supermarket chains are also likely to use much more sophisticated indicators of the characteristics of the local area and potential customers, thus applying one indicator of area-level socioeconomic disadvantage may not be adequate to determine whether stocking practices differ between areas.

This study is strengthened by the use of an audit tool that captured a more detailed display of snack food availability than that measured in previous studies. By measuring checkouts, end-of-aisle displays and island bin displays, we captured the deliberate placement of dynamic displays that are designed to increase impulse purchases of snack foods made by customers. Whilst we undertook a small amount of test-retest reliability audits, we recognise that our analysis relies on a single within-store observation. Our reliability tests suggested some small variation in the products being offered in the end-of-aisle and island bins but on closer examination these variations were from one snack food product to another (e.g. from chocolate to soft drink) rather than from a snack food product to a non-snack food product. Further, a previous study from the US that examined produce within-store suggested that stores have at least short-term stability and that a single observation is often an accurate reflection of the stores' usual stocking practices [31]. The snack foods included in our study are not the only energy-dense, nutrient poor foods available to customers within supermarkets. A universal definition of 'snack food' does not exist [32,33], and for the context of this research, we limited our definition to food and beverage types that are often consumed outside of the three main meals and would be considered energy-dense, high in sodium and low in micronutrients.

## Conclusions

This study found evidence of extensive snack food availability at checkouts, in end-of-aisle displays and in island displays within supermarkets. By simultaneously exploring multiple exposures we demonstrate that consumers have very little chance of avoiding snack food displays in Melbourne supermarkets. Research findings of this nature are a necessary first step to quantify and raise awareness of unhealthy environmental exposures and can inform coalitions that are engaged in promoting public health. Initiatives aimed at limiting the availability of displays of unhealthy items within-stores are urgently required.

## Competing interests

The authors declare that they have no competing interests.

## Authors' contributions

LT and AC drove the conceptualisation of the study design, development of the audit tool, undertook the analysis, and wrote the first draft of this paper. SMc, AW, and DC contributed to the study design and redrafting of the paper. Each author has read and approved the final version of this manuscript.

## Pre-publication history

The pre-publication history for this paper can be accessed here:

http://www.biomedcentral.com/1471-2458/12/194/prepub
